# Are Risky Choices Actually Guided by a Compensatory Process? New Insights from fMRI

**DOI:** 10.1371/journal.pone.0014756

**Published:** 2011-03-11

**Authors:** Li-Lin Rao, Yuan Zhou, Lijuan Xu, Zhu-Yuan Liang, Tianzi Jiang, Shu Li

**Affiliations:** 1 Key Laboratory of Behavioral Science, Institute of Psychology, Chinese Academy of Sciences, Beijing, China; 2 Sino-French Laboratory for Computer Science, Automation, and Applied Mathematics (LIAMA) and National Laboratory of Pattern Recognition, Institute of Automation, Chinese Academy of Sciences, Beijing, China; University of Granada, Spain

## Abstract

The dominant theories about risky decision-making assume that decision conflicts are solved by a compensatory process involving a trade-off of probability against payoff, but it is unclear whether these theories actually represent the events that occur when people make a risky decision. By contrasting a preferential choice with a judgment-based choice that required a compensatory process, we explored the mechanisms underlying risky decision-making. First, using parametric analyses, we identified the dorsomedial prefrontal cortex (dMPFC) as the specific region in charge of task-related conflict in risky decision-making tasks. We also showed that the dMPFC was activated less when judgment-based choices were being made, implying that the conflict experienced during a judgment-based choice was not as strong as the conflict that was experienced during the preferential choice. Our results provide neural evidence that preferential choice cannot be characterized solely as a compensatory process. Thus, questions were raised about whether existing compensatory theories could adequately describe individual risky decisions.

## Introduction

Risky decisions become difficult when the payoff and probability are in conflict. Mainstream theories of decision-making under risk, from expected utility theory [1] to prospect theory [2], [3], [4], assume that the problem of decision conflict can be solved by a compensatory process that trades probability for payoff. Thus, the attractiveness of a bet offering payoffs (*x_1_*, …, *x_n_*) with probabilities (*p_1_*, …, *p_n_*) is given by its mathematical expectation 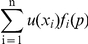, where *u* denotes the utility function *f_i_*(*p*) for vector *p* and different functions indexed by *i* take appropriate elements of vector *p* and weight them. This idea seems simple and straightforward because decision-makers are only required to compute the mathematical expectation of each alternative with respect to function *f* before choosing the option that maximizes overall value or utility.

This assumption has led neuroscientists to attempt to identify the neural basis for each of the components that are required to produce an overall value or utility. Neuroimaging studies have identified potential neural substrates associated with payoff and probability [5], [6], . Neural structures reported to be activated in a representation of a payoff include the orbitofrontal cortex, insula, thalamus and striatum [9], [10], [11], [12], [13]. Activations of the lateral prefrontal cortex (PFC), the posterior parietal cortex (PPC), and the insular cortices have been correlated with probability in previous studies [6], [14], [15], [16], [17], [18]. Specifically, researchers have suggested that the dorsolateral prefrontal cortex might play a role in risky decision making by computing decision utility [8].

However, it remains unclear whether the compensatory model actually represents what happens when people make a risky decision. The processes of weighing and summing have been challenged before (e.g., [19], [20], [21]). Researchers have argued that an individual's preferential choice can be better described by a non-compensatory process, such as the lexicographic semi-orders, equate-to-differentiate, priority heuristic, or single-dimension heuristics [19], [22], [23], [24], [25], [26]. Non-compensatory models do not allow deficiencies in one attribute to be compensated for by high values from another attribute. Behavioral evidence exists that people use only one dimension (either probability or payoff) at a time to reach a decision [23], [27]. Neural evidence also exists for separate neural mechanisms underlying different strategic preferences in risky decision making [28], [29]. The dorsomedial prefrontal cortex (dMPFC) has been reported to play a strategy-related role in risky decision-making [8], [29].

The debate about whether risky choices are based on a compensatory process or a non-compensatory process has a long history. Recently, the *Psychological Review* published a series of papers on this issue [26], [30], [31], [32], [33], [34], [35], [36]. Using behavioral data, Brandstätter, Gigerenzer and Hertwig contended that their non-compensatory model was more accurate than compensatory theories [23], [26], [33]. However, other researchers have also used behavioral data to support compensatory theories [30], [32], [34], [35], [36]. Thus, to date, behavioral data have not been able to resolve this controversy.

The primary difference between compensatory processes and non-compensatory processes appears to be associated with the differences in the solutions to conflicts between binary alternatives [37]. According to the compensatory rule, individuals are forced to weigh and sum all of the possible outcomes to assign a unidimensional value to each alternative, thereby exposing the individuals' preferences [38]. One alternative comes to dominate the other along the unidimension, and the conflict is resolved [20]. In this compensatory process, a stronger conflict induced by maximization would result when less of a difference was determined to exist between the overall values or the utilities of two alternatives on the unidimension alone. Non-compensatory rules, on the other hand, forgo weighing and summing. Instead, these rules eliminate any alternative that has a low value in one dimension, even if that alternative rates highly in the other dimension. Because the decision is not based on a unidimensional value, no alternative would strongly dominate the others in terms of overall attributes. When people are unable to find a dominant alternative, they experience conflict [26]. In this non-compensatory process, a higher conflict would be related to greater intradimensional differences between the two alternatives for both dimensions (*i.e.*, payoff and probability).

Because few behavioral indices for monitoring internal conflict have been described [39], and because behavioral indices might not directly reflect the internal process, researchers have not been able to use behavioral data to resolve whether risky choices are based on compensatory or non-compensatory processes in situations where internal conflict exists. Considering that neuroimaging may be useful for detecting internal conflict, we conducted a functional magnetic resonance imaging (fMRI) study to explore generic risk-related processing. Keeping in mind the distinct differences between compensatory and non-compensatory processes in conflict situations, we focused on the neural basis of conflict monitoring by contrasting a judgment-based choice task with a preferential choice task. The difference between the two tasks was that the judgment-based choice task required participants to perform a compensatory process of trading off probability against payoff by using the certainty equivalent method [40], [41], but the preferential choice task did not explicitly require participants to perform a compensatory or non-compensatory process.

Using the two factors (i.e., the unidimensional difference between overall values or utilities of two alternatives on the unidimension alone and the intradimensional difference between two alternatives on the payoff/probability dimension) as the parameters for separate parametric analyses, we identified the conflict-related brain region in our study of risky decision making. Then, we showed differential neural sensitivities to these parameters between the preferential choice and the judgment-based choice. Finally, we showed evidence that the intensity of inner conflict revealed by our identified conflict-related region was less pronounced in the compensatory choice (i.e., the judgment-based choice) than in the preferential choice. These neural measurements will help us to better understand the processes that underlie compensatory and non-compensatory decision making and to address the question of whether risky choices are guided by a compensatory process as assumed by expected utility theory and its extensions.

## Materials and Methods

### Participants

Twenty-six undergraduate or graduate students (11 males, mean age 22 years, SD 2.81) participated in this study. Three participants were excluded from the analyses because of excessive head motion. All participants were in good health with no previous history of psychiatric or neurological disease, and they gave written informed consent. The study was approved by both the Institutional Review Board of the Institute of Psychology, the Chinese Academy of Sciences, and the Institutional Review Board of the Beijing MRI Center for Brain Research.

### Experimental design and task

**Figure 1:**
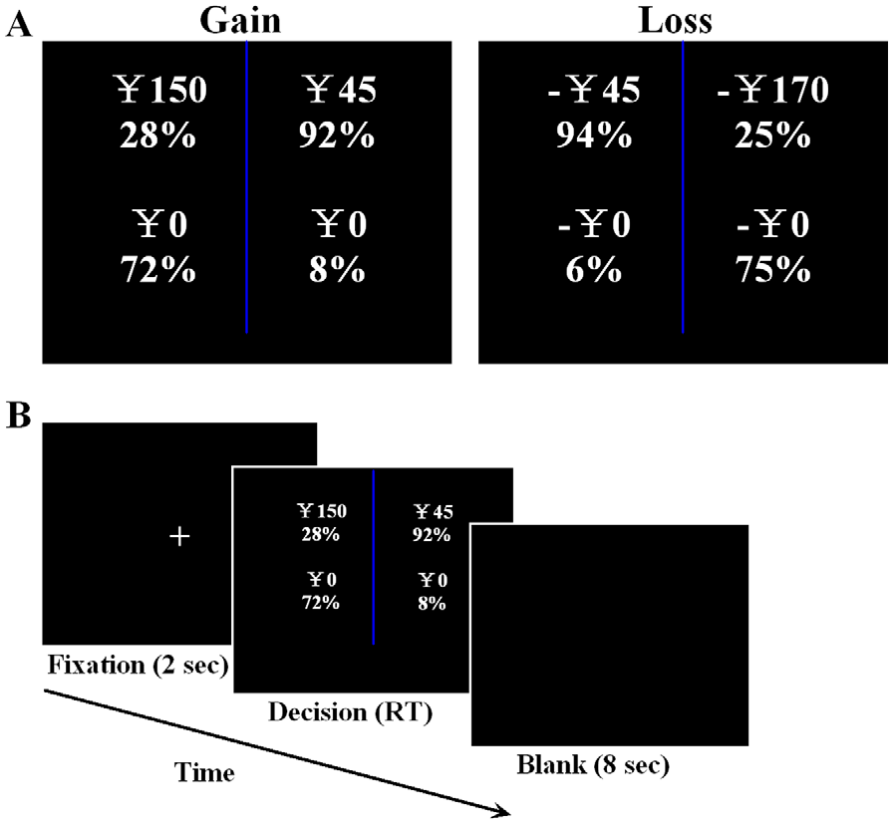
Task trial structure. A. Trial types. B. Trial timing. Two bets were presented and the participants' task was to indicate their decision by pressing one of two buttons on a response pad.

We constructed 60 pairs of two-outcome monetary bets: one featuring a high probability of winning/losing a modest amount of money (the P bet), and the other featuring a low probability of winning/losing a relatively large amount of money (the $ bet) (Figure 1A). Outcome probabilities of the P bet ranged from 81% to 97% in 2% or 3% increments, and the outcome probabilities of the $ bet ranged from 19% to 39% in 2% or 3% increments. The expected values for the P bets and the $ bets ranged from Chinese Yuan (CNY) ±16 to ±44.

In the preferential choice task, participants were asked to select their preferred bet from each pair. To make the participants perform a compensatory process, we developed a judgment-based choice task using the certainty equivalent method [42], [43]. This method has traditionally been based on expected utility theory [41]. This technique uses a lottery to assess a decision maker's preferences over a single attribute (which was either probability or payoff) range and trade-offs [40]. In the judgment-based choice task, participants were asked 1) to use the certainty equivalent method to assess the certain gain/loss equivalent of each bet (in other words, the cash equivalent that the participants felt would make them indifferent to the given bet), and 2) the participants were asked to select the bet with the higher-certainty gain/loss equivalent from each pair. Participants did not explicitly indicate the certainty equivalent for each bet before indicating their choice. In this manner, a compensatory process of trading off probability against payoff was performed. The order of the tasks was counterbalanced between the participants. In each of the preferential and judgment-based choice tasks, half of the participants were assigned to view the gain trials before the loss trials, and the other half viewed the trials in the opposite order. The order of the trials within each domain was randomized, and the order of domain was counterbalanced between the two tasks and within the participant group.

Each participant performed both the preferential and judgment-based choice tasks with an interval of at least 7 days between the two tasks. Prior to entering the scanner, the participants played a practice version of either the preferential choice task or the judgment-based choice task to minimize learning effects during the actual scanning and to enable them to fully understand the paradigm. During the scanning, the participants were asked to perform the tasks with no time constraints. They made their decisions by pressing one of two buttons corresponding to the location of the bets on the screen. After the participants' response, there was a delay of 8 s before a 2 s fixation procedure started and indicated the next bets (Figure 1B). There was a 10-min interval between the gain trials and loss trials for each participant. During this period, high-resolution structural images were acquired, but these data were not used in this study. After the scan, participants were asked whether they integrated the probability and the payoff to estimate the certainty equivalent for each bet by using a method similar to the mathematical expectation in the judgment-based choice task. Twenty-one of twenty-three participants reported that they integrated the probability and the payoff to estimate the certainty equivalent for each bet using a method similar to mathematical expectation. The two negative answers came from participants who claimed that they did not know about mathematical expectation. Participants were also checked to ensure that they had been fully engaged in each task and clearly understood the tasks. All participants reported a belief that they would win or lose money at the end of the tasks. They were told that at the end of each task, two of their decisions would be randomly selected to be played for real (one in the gain domain and the other in the loss domain), and another random device would determine the outcomes of the selected bets. Both of the random choices were performed by a MATLAB program. If the result turned out to be a gain, the participants would receive money from the experimenter. If the result turned out to be a loss, the participants would give money to the experimenter or sign an IOU if they could not afford to make a cash payment. The average real gain over the two selected bets was ¥29.57 per participant, whereas the average real loss over the two selected bets was -¥31.52 per participant. At the completion of the study, the participants were paid ¥100 in cash for participating, and the debts/winnings determined by the above method were deducted from/added to the final payment.

### fMRI acquisition

MR images sensitized to changes in BOLD signal levels were obtained by an echo planar imaging sequence on a 3.0-Tesla Siemens MR scanner (repetition time = 2,000 msec; echo time = 30 msec; flip angle = 90°, matrix = 64×64; field of view = 220×220 mm^2^; slice thickness = 3 mm; slice gap = 1 mm and, thus, acquisition voxel size = 3.4×3.4×4 mm^3^). Each brain volume was composed of 32 axial sections. Stimuli were presented with E-prime software (Psychology Software Tools, Pittsburgh, PA) on a personal computer, back-projected onto a screen using a liquid crystal display projector and viewed by participants through a mirror mounted on the MRI head coil. We also acquired a brief (6-min) resting-state scan, which was composed of 180 volumes (the data were not used in this study).

### fMRI preprocessing and analyses

Image preprocessing and analyses were performed using statistical parametric mapping (SPM5, Wellcome Department, London, UK) that was run on a MATLAB 7 platform (MathWorks, Natick, MA). The first three volumes in each scan series, which were collected before equilibrium magnetization was reached, were discarded. The remaining images were corrected for within-scan acquisition time differences between the slices and then realigned to the first volume to correct for inter-scan head motions. Based on a visual inspection of the motion correction estimates, three participants who had more than 2-mm maximum displacement in any of the x, y or z directions or more than 2° of angular rotation about any axis for multiple volumes were excluded from this study. An additional two participants showed sudden head motion only in the first trial, which allowed the use of the imaging data obtained from these two participants after the first trial was removed. The realigned images were spatially normalized to the standard EPI template, resampled to 3×3×3 mm^3^ and smoothed using an 8-mm full-width-at-half-maximum Gaussian kernel to decrease spatial noise. Motion parameters were stored and used as nuisance variables in the following generalized linear model (GLM) analysis.

Individual participant data were analyzed using the general linear model (GLM) with separate models for each task. Events were modeled with a variable-duration boxcar function convolved with a canonical hemodynamic response function (HRF). To quantitatively describe the relationship between brain activation and decision parameters, parametric analyses were performed.

Trials were modeled with parametric regressor modeling (in the following order): (1) the intradimensional difference in the probability dimension or in the payoff dimension, and (2) the unidimensional difference between overall values/utilities of two bets on the unidimension alone. To avoid a choice in which one bet dominated the other, all of the bets used in our study were designed to be choices between high payoffs with low probabilities and low payoffs with high probabilities. As a consequence, we observed a significant correlation (*r = *0.483, *p* = 0.007) between the differences on the payoff dimension and the differences on the probability dimension. Thus, an increase in the difference in the payoff dimension would result in an increase in the difference in the probability dimension. To avoid the problems induced by severe co-linearity, we chose one intradimensional difference to enter into the regressor model at a time. Therefore, there were two parametric analyses for each task. For example, Model I was analyzed for the intra-dimensional difference in the probability dimension and the unidimensional difference, and Model II was analyzed for the intradimensional difference in the payoff dimension and the unidimensional difference.

To estimate the intradimensional difference in the payoff dimension and the intradimensional difference in the probability dimension, we calculated the utilities for the objective payoff and the decision weights for the objective probability separately. The utility function and the probability weighting function were borrowed from cumulative prospect theory [4], which is a compensatory theory that is considered to be empirically successful [3]. The selected utility function was the power function 
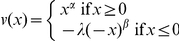
, and the selected probability weighting function was an S-shaped weighting function, i.e., 
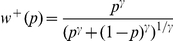
 for gains and 
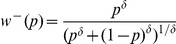
 for losses. We used the following values for each of the parameters: α = β = 0.88, λ = 2.25, γ = 0.61 and δ = 0.69, as suggested by Tversky and Kahneman [4].

To estimate the unidimensional difference between the overall values/utilities of two bets in the unidimension alone, we calculated the cumulative prospect utilities of the two bets by applying the function of cumulative prospect theory 
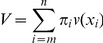
 [4]. According to cumulative prospect theory, the utility function is 
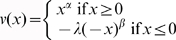
, and the probability weighting functions are 
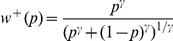
 for gains and 
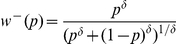
 for losses. The function of overall utility is 
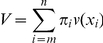
, where 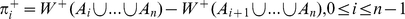 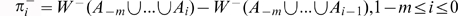.

All effects were modeled as linear functions of parameter values. Regressors were serially orthogonalized with the regressors that were entered later, accounting only for the variance unaccounted for by regressors that were entered earlier. Participant-specific movement parameters and linear drift were modeled as covariates of no interest. A high-pass filter with a cutoff period of 128 s was used to remove low-frequency noise. Global scaling was not applied.

First-level (single participant) contrasts were performed separately over the mean trial activity (relative to baseline) and for each of the parametric regressors described above. Second-level (group) random effect analyses were completed by performing one-sample t-tests over each of these contrasts. Corrections for multiple comparisons were carried out at the cluster level using Gaussian random field theory implemented in SPM5 (min T(22)>2.5; cluster significance: *p*<0.05, corrected). By using the intradimensional difference in the probability/payoff dimension and the unidimensional difference between the cumulative prospect utilities of two bets as parameters, parametric analyses were conducted to identify brain regions that were sensitive to intradimensional difference and unidimensional difference. Paired t-tests were used to further verify the differences in the sensitivity to the specific decision parameters (*p*<0.005, uncorrected). To control for the difference in task difficulty between the two tasks, the average RT of each participant within each task was taken as an index of task difficulty and entered into the paired t-test analysis as a covariate.

Finally, a mask composed of the conflict-related regions that were sensitive to the unidimensional difference and the intradimensional difference in the payoff or probability dimension was generated. According to our hypothesis, the conflict-related regions should be those regions in which the unidimensional difference between the two offered bets shows a negative modulation effect or regions in which the intradimensional difference on the probability/payoff dimension shows a positive modulation effect (min T(22)>2.5; cluster significance: *p*<0.05, corrected). Thus, a mask was formed in which each voxel value was set to one if the voxel satisfied one of the above criteria. The mask was set to zero if the criteria were not satisfied. To compare activities (relative to baseline) in the conflict-related regions between the two tasks, another random-effect paired t-test analysis controlling for the effect of task difficulty was used to determine where the second-level group contrasts differed between the preferential choice task and the judgment-based choice task. This analysis was limited to the mask (*p*<0.005, uncorrected).

## Results

### Behavioral results

**Figure 2:**
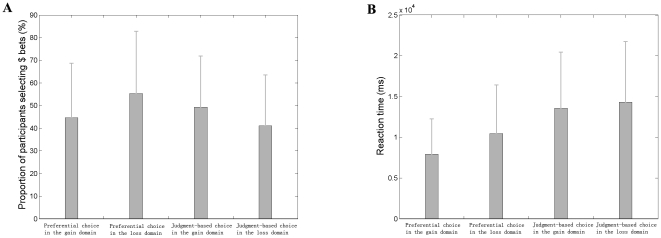
Behavioral results. A. Mean proportion of participants selecting $ bets. B. Reaction time as a function of task. The error bars denote the standard deviation.

Behavioral results are shown in Figures 2A and B. We conducted a 2 (task: preferential choice vs. judgment-based choice) × 2 (domain: gain vs. loss) repeated-measures ANOVA to compare the mean differences in the proportion of participants selecting the $ bets. The results revealed that there were no main effects of task (*F*(1, 22) = 1.29, *p*>0.05) or domain (*F*(1, 22) = 0.031, *p*>0.05). The interaction between task and domain was statistically significant (*F*(1, 22) = 4.75, *p*<0.05). A simple effect test indicated that in the loss domain, participants selected more $ bets in the preferential choice task than in the judgment-based choice task (*p*<0.05).

We also conducted an ANOVA to compare the reaction time difference between the two tasks. Analyses of reaction time revealed that the participants took more time to make a judgment-based choice than to make a preferential choice (*F*(1, 22) = 10.90, *p*<0.01). They also took more time to make a decision in the loss domain than in the gain domain (*F*(1, 22) = 7.77, *p*<0.05). The interaction between task and domain was not significant (*F*(1, 22) = 2.32, *p*>0.05).

Hierarchical multiple regression analyses were then conducted to examine the influence of the intradimensional and unidimensional differences between the two bets on the absolute difference between the proportion of participants selecting the $ bet and the proportion of participants selecting the P bet in each pair (hereafter, proportion selecting difference) in two tasks. In the first block, the intradimensional difference in the probability dimension and in the payoff dimension were entered into the analysis using the enter method. In the second block, unidimensional differences between overall values/utilities of two bets were included using the stepwise method because it can identify those factor(s) for which the significance level is less than 5%. The included unidimensional differences between the two bets were derived from expected value (EV) theory, expected utility (EU) theory (where U = ln(x) or U = lg(x)), and the cumulative prospect theory (where α = 0.88, λ = 2, γ = 0.61, δ =  0.69, or α = 0.88, λ = 2.25, γ = 0.61, δ = 0.69).

For the preferential choice task, all of the unidimensional differences between overall values/utilities of two bets were excluded as predictors from the final regression model. There was an independent negative relationship between the intradimensional difference in the payoff dimension and the proportion selecting difference between the two bets (*t* = −2.019, standardized beta = −0.371, *p*<0.05), implying that a larger difference in the payoff dimension could effectively induce a smaller proportion selecting difference between the two bets (i.e., participants chose more evenly). There were no other significant effects (all absolute *t*≤1.436, all absolute standardized beta≤0.264, all *p*≥0.157).

For the judgment-based choice task, inclusion of the unidimensional differences in expected values, cumulative prospect utilities (where α = 0.88, λ = 2, γ = 0.61, δ = 0.69), and cumulative prospect utilities (where α = 0.88, λ = 2.25, γ = 0.61, δ = 0.69) in the second block produced a significant F-change (*F* = 7.343, *p*<0.001, adjusted R^2^ = 0.350), while the unidimensional differences between expected utilities (where U = ln(x) or U = lg(x)) were excluded as predictors from the final regression model. In the final model, the unidimensional difference in cumulative prospect utilities (where α = 0.88, λ = 2.25, γ = 0.61, δ = 0.69) (*t* = 2.424, standardized beta = 5.437, *p*<0.05) and in expected values (*t* = 0.501, standardized beta = 3.916, *p*<0.001) showed positive relationships with the proportion selecting difference, while the unidimensional difference in cumulative prospect utilities (where α = 0.88, λ = 2, γ = 0.61, δ = 0.69) showed a negative relationship with the proportion selecting difference (*t* = −2.709, standardized beta = −5.599, *p*<0.01). The fact that the standardized beta coefficient of cumulative prospect utilities (where α = 0.88, λ = 2.25, γ = 0.61, δ = 0.69) was the largest positive coefficient suggests that the greater unidimensional difference (i.e., the greater difference in cumulative prospect utilities where α = 0.88, λ = 2.25, γ = 0.61, δ = 0.69) between the two bets could effectively induce a greater proportion selecting difference between the two bets (i.e., participants chose less evenly).

### Neuroimaging results

**Figure 3:**
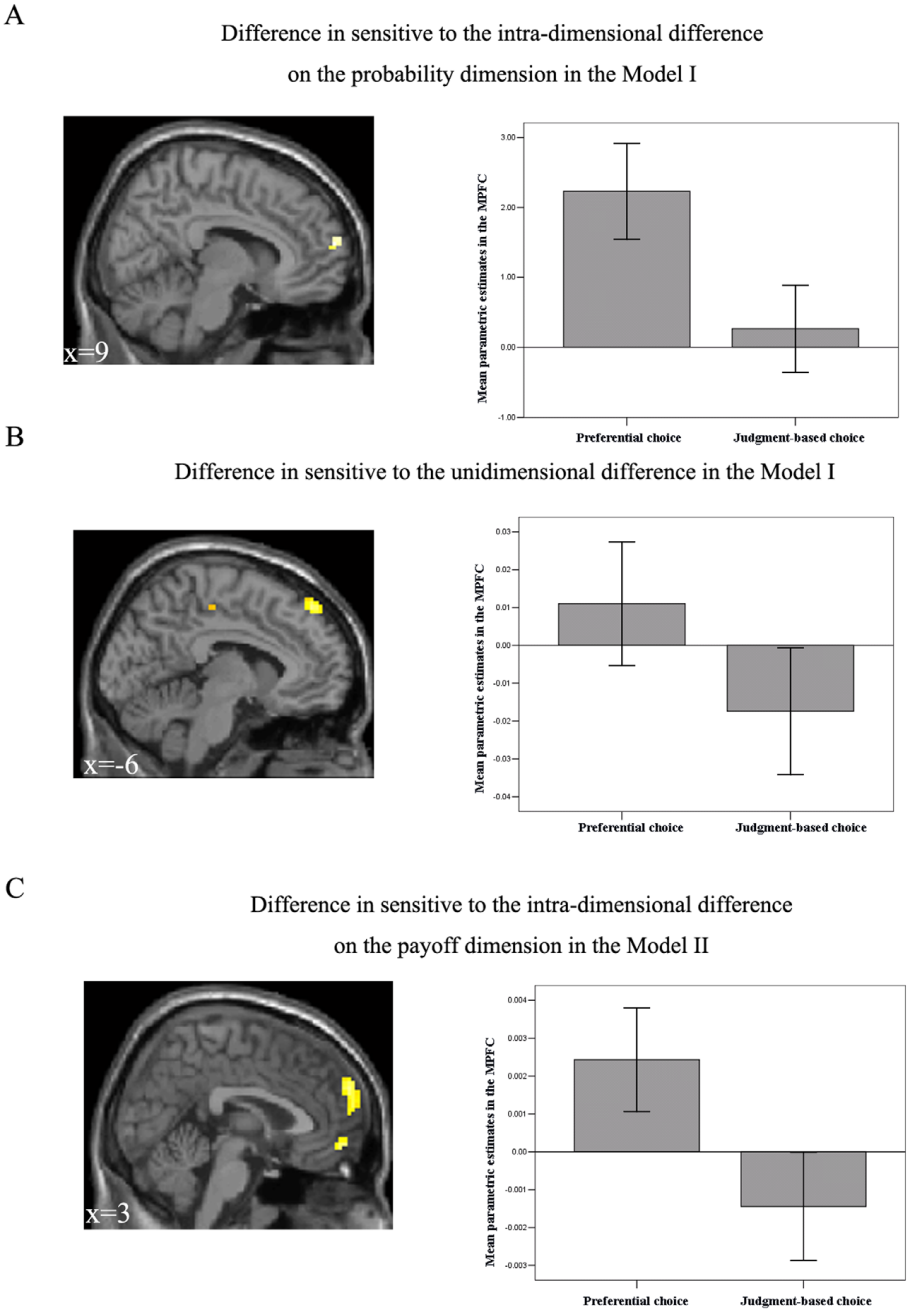
Brain regions tracking variations in decision parameters. MPFC showed differences in sensitivity to the intradimensional difference in the probability dimension in Model I (A), in sensitivity to the unidimensional difference in Model I (B), and in sensitivity to the intradimensional difference in the payoff dimension in Model II (C). In the left column, the region was displayed in a T1-weighted MRI template. In the right column, the mean parameter estimates of the corresponding region in the two choice tasks were shown.

We performed whole-brain analyses to identify brain regions exhibiting parametric increases or decreases in BOLD signals while specifically tracking changes in the intradimensional difference and the unidimensional difference. In the parametric regressor model with the intradimensional difference in the probability dimension and the unidimensional difference between the cumulative prospect utilities of two bets as parameters (Model I), a positive correlation with the intradimensional difference was observed in the medial prefrontal cortex (MPFC) that extended to the anterior cingulate cortex (ACC)., and no opposite effects were observed in the preferential choice task. This positive modulation effect meant that the activity of the region increased when the difference in the probability dimension increased, just as we hypothesized. However, in the judgment-based choice task, no regions were sensitive to the intradimensional difference in the probability dimension (min T(22)>2.5; cluster significance: *p*<0.05, corrected) (Table 1). A negative correlation with the unidimensional difference was observed in the dMPFC, the left temporal regions and the bilateral visual cortices. No opposite effects were observed in the judgment-based choice task. This negative modulation effect meant that the activity of the region increased when the difference between the cumulative prospect utilities of two bets decreased. In the preferential choice task, however, no regions were sensitive to the unidimensional difference (min T(22)>2.5; cluster significance: *p*<0.05, corrected) (Table 1). Paired t-tests controlling for the effect of task difficulty further verified the differences in the sensitivity to the parameters. In brief, we found that the dMPFC showed both decreased sensitivity to the intradimensional difference in the probability dimension (MNI spatial coordinates of peak voxel: x = 9, y = 63, z = 15, T = 4.03) (Figure 3A) and increased sensitivity to the unidimensional difference in the judgment-based choice task compared to the preferential choice task (MNI spatial coordinates of peak voxel: x = −6, y = 45, z = 51, T = 4.26) (*p*<0.005, uncorrected) (Figure 3B).

**Table 1:** Regions sensitive to the intradimensional difference in the probability dimension and regional sensitivity to the unidimensional difference within each task.

Regressor	Cluster size	Region	BAs	Peak MNI coordinates	Peak T value
**Intradimensional difference in the probability dimension**					
	**Preference choice task**				
	*Positive modulation*				
	1781	Bilateral dorsal and ventromedial MPFC extending to ACC	6/8/9/10/11/24/32	0 66 27	6.66
	*Negative modulation*				
	none				
	**Judgment-based choice task**				
	none				
**Unidimensional difference on the CPT utility**					
	**Preference choice task**				
	none				
	**Judgment-based choice task**				
	*Positive modulation*				
	none				
	*Negative modulation*				
	737	Left inferior and middle temporal gyrus	20/21/37	−45 −45 −15	5.14
	2582	Bilateral visual cortex and cerebellum extending to posterior cingulate cortex		21 −105 −6	5.1
	621	Bilateral dMPFC extending to the right superior and middle frontal gyrus		−27 21 48	4.23

Similarly, in the parametric regressor model with the intradimensional difference in the payoff dimension and the unidimensional difference between the cumulative prospect utilities of two bets as parameters (Model II), a positive correlation with the intradimensional difference in the payoff dimension was observed in the MPFC in the preferential choice task (min T(22)>2.5; cluster significance: *p*<0.05, corrected). However, in the judgment-based choice task, no regions were sensitive to the intradimensional difference in the payoff dimension (min T(22)>2.5; cluster significance: *p*<0.05, corrected) (Table 2). A paired t-test controlling for the effect of task difficulty further verified the differences in the sensitivity to this intradimensional difference by finding decreased sensitivity to this parameter in the MPFC in the judgment-based choice task compared to the preferential choice task (min T(22)>2.5; cluster significance: *p*<0.05, corrected) (MNI spatial coordinates of peak voxel: x = 3, y = 60, z = 30, T = 3.89) (Figure 3C). A negative correlation with the unidimensional difference was observed in the bilateral visual cortices (min T(22)>2.5; cluster significance: *p*<0.05, corrected) and the MPFC (which survived the height but not the extent threshold) in the judgment-based choice task but not in the preferential choice task. Although the pattern in sensitivity to the unidimensional difference within each task was similar to the sensitivity in Model I, the differences between the two tasks in this model (Model II) were not significant in the MPFC even under an uncorrected threshold of *p*<0.01.

**Table 2:** Regional sensitivity to the intradimensional difference in the payoff dimension and regional sensitivity to the unidimensional difference within each task.

Regressor	Cluster size	Region	BAs	Peak MNI coordinates	Peak T value
**Intradimensional difference on the payoff dimension**					
	**Preference choice task**				
	*Positive modulation*				
	379	Bilateral dMPFC		12 63 27	4.77
	*Negative modulation*				
	none				
	**Judgment-based choice task**				
	none				
**Unidimensional difference on the CPT utility**					
	**Preference choice task**				
	none				
	**Judgment-based choice task**				
	*Positive modulation*				
	none				
	*Negative modulation*				
	2008	Bilateral visual cortex and cerebellum		18 −87 −9	6.01
	56	Left ventromedial MPFC*	10	−15 54 −9	3.68
	50	Left dMPFC*	10	−15 36 33	3.34

*Two clusters located in the left MPFC survived the height but not the extent threshold.

Finally, we compared activities (relative to baseline) in the conflict-related regions between the two tasks to test whether the intensity of inner conflict was less pronounced in the judgment-based choice than in the preferential choice. Based on the parametric analyses, the conflict-related regions that were sensitive to the unidimensional difference and the intradimensional difference in the probability/payoff dimension were obtained. Because the BOLD activities (relative to baseline) in the Model I were exactly the same as the activities in Model II and because both models were based on the same task regressor, we only showed the results from Model I. Using Model I, the dMPFC showed increased activity in the preferential choice task compared to the judgment-based choice task (*p*<0.005, uncorrected) (Table 3). No regions showed decreased activity in the preferential choice task compared to the judgment-based choice task.

**Table 3:** Regions showing differences in activation between the preferential choice task and the judgment-based choice task.

Cluster size	Region	BAs	Peak MNI coordinates	Peak T value
**Preferential choice task > Judgment-based choice task**				
69	Left inferior and middle temporal gyrus	37	−48 −36 −12	4.23
18	Left inferior temporal gyrus	21	−42 3 −36	3.71
12	Right dMPFC	10	6 66 24	3.61
14	Left dMPFC	10	−15 66 24	3.39
**Preferential choice task > Judgment-based choice task**				
none				

## Discussion

Understanding the underlying mechanism of decision making under risk remains a fundamental and difficult problem. Although there has been much debate about whether risky choice is based on compensatory or non-compensatory processes, to our knowledge, nothing has previously emerged to give decision analyst a pause about using the compensatory rule as the fundamental principle of decision making. The family of compensatory theories has kept its dominant position, in part through continuing generations of prospect theory [2], [3], [4], which is a theory that has been widely accepted as empirically successful. The present study, however, provided several interesting results with implications for the underlying mechanisms of risky choices. We found that (1) high-probability bets tended to be avoided in the loss domain in the preferential choice task, but this tendency decreased in the judgment-based choice task; (2) participants took more time to make a judgment-based choice than to make a preferential choice; (3) participants chose more evenly as the unidimensional difference between the two bets decreased in the judgment-based choice task, whereas participants chose more evenly as the intradimensional difference between two bets increased in the preferential choice task; (4) the dMPFC activation increased as the unidimensional difference between the two bets decreased in the judgment-based choice task, whereas activation increased as the intra-dimensional difference in the payoff dimension increased in the preferential choice task; (5) the dMPFC showed more activity during the preferential choice task than during the judgment-based task. These results generally supported our hypothesis that preferential choice may not be a purely compensatory process.

### What (and how long) to choose?

The choice result revealed that our participants tended to choose one member of the pair but set a higher certainty equivalent for the other pair. However, the observations made about preferential choice could still be interpreted by compensatory proponents as being guided by a compensatory rule. For instance, the contingent weighting model [42] and third-generation prospect theory [3] were developed to reconcile compensatory processing with the preference reversal phenomenon.

Previous research also reported that compensatory processing takes more time than non-compensatory processing [43], [44] because the decision-making mechanism is a two-stage process in compensatory processing. According to the compensatory models of risky choices, individuals have to compute the mathematical expectation first and then choose the option with the higher overall value or utility. Note that if a decision maker selects an option with the greatest overall value or utility (presumably determined by the certainty equivalent method) in a preferential choice, the judgment-based and preferential tasks should employ the same decision-making process and, presumably, require the same amount of time. The finding that the participants took more time to make a judgment-based choice than to make a preferential choice suggests that our preferential choice task may employ a process besides compensation.

A novel behavioral finding was that the intradimensional difference and the unidimensional difference influenced preferential choice and judgment-based choice, respectively, in terms of the absolute difference between the proportion of participants selecting the $ bet and the proportion of participants selecting the P bet in each pair. Such a finding suggests that the impact of intradimensional versus unidimensional differences between two bets is task dependent (task specific). Thus, preferential choice is influenced by intradimensional differences but not by unidimensional differences between two bets, whereas judgment-based choice is influenced by unidimensional differences but not by intradimensional differences between two bets. Notably, an even selection (50:50) without strong preference for either bet indicates that participants were unable to detect a dominance relationship between the bets and thus experienced great conflict.

### Which region is in charge of conflict monitoring in risky decision making?

Cognitive neuroimaging and neuropsychology studies have consistently shown that the ACC/dMPFC, which has been widely interpreted to respond to the presence of conflict [28], [29], [45], [46], [47], is critically active in a wide variety of cognitive settings. In particular, the ACC/dMPFC has been suggested to be a monitoring center from which a conflict signal detected by the ACC/dMPFC is transmitted to other brain regions [45]. However, the conflict-related regions in these studies have not been precisely pinpointed in relation to risky decision making. Moreover, it should be noted that other interpretations of the functional role of ACC/dMPFC have been developed, such as error likelihood [48], [49], regret [50], action value [51], [52], response evaluation [53], and uncertainty [54], [55], [56].

Considering that the functional role of the ACC/dMPFC might be context-specific [55], we attempted to identify whether the activity of ACC/dMPFC could be related to conflict monitoring in our study using parametric analyses. The result of this independent study revealed that the activation of the dMPFC showed great sensitivity to the unidimensional difference and the intradimensional difference. In the judgment-based choice, the activation in the dMPFC was highest when the cumulative prospect utilities of the two bets were closest. However, in the preferential choice task, the activation was the highest when the intradimensional difference between the two bets was the highest. These findings combined with the proportion selecting difference results led us to consider that the dMPFC was in charge of task-related conflict in the tasks performed in this study. This result is consistent with those of a recent study published by Venkatraman and colleagues (2009), which showed that similar regions play an important role in decision conflict [28].

As our interpretations were made under the very particular conditions of risky decision making tasks, this interpretation should be regarded as tentative until further work excludes alternative interpretations.

### How to solve conflict?

Guided by the different theoretical orientations, researchers have proposed different possible resolutions to conflict from the compensatory and non-compensatory points of view. In the debate between the compensatory and non-compensatory models, various behavioral methods, ranging from off-line questionnaires [23] to Mouselab [34], have been used. However, the extant behavioral literature appears to be inconclusive. Our study, therefore, was an attempt to overcome this difficulty by examining behavioral and neural data and their relation to the bet parameters.

When there was a choice available between high payoffs with low probabilities and low payoffs with high probabilities, the participants seemed to employ a less mathematically complex strategy to make decisions in the preferential choice situation because these decisions were associated with faster response time. More importantly, the hierarchical multiple regression analyses suggested that participants used different strategies to make decision in two tasks. In the preferential choice task, increased proportion selecting difference was associated with a decrease in the difference in the payoff dimension, implying that the conflict pattern (in terms of the proportion selecting difference) in the preferential choice resulted from using a non-compensatory process without trading off probability against payoff. In the judgment-based choice task, on the other hand, increased proportion selecting difference was associated with an increase in the unidimensional difference (estimated using cumulative prospect theory), implying that the conflict pattern in the judgment-based choice was induced by the use of a compensatory process of trading off probability against payoff. Furthermore, we found a clear difference in the activation of the dMPFC between the responses to the two parameters in the two different tasks. The parametric analyses revealed that the parameters of the unidimensional difference (estimated using cumulative prospect theory) were poor predictors of dMPFC activation in the preferential choice but were good predictors of dMPFC activation in the judgment-based choice. In contrast, the parameters of the intradimensional difference (payoff and probability separately) were good predictors of the dMPFC activation in the preferential choice, but they were poor predictors of dMPFC activation in judgment-based choice. These results provided behavioral and neural evidence that preferential choice is non-compensatory, or at least not purely compensatory.

To the extent that dMPFC activity has been identified as playing an important role in conflict monitoring, our finding that dMPFC activation increased during the preferential choice task suggests that the conflict participants experienced during the judgment-based choice was not as strong as the conflict experienced during the preferential choice. This finding strengthens our doubt that individuals were seeking a dominant alternative as a means of solving decision conflict by using the weighing and summing process that was suggested by a compensatory rule.

A limitation of our study is that we only completed a global check rather than a trial-by-trial check by asking participants to explicitly indicate the certainty equivalence for each bet during scanning. There is a possibility that participants' certainty equivalence estimate may vary for repeated attempts to estimate the value of a gamble in the judgment-based choice task. Although the longer RT and the positive relationship between the proportion selecting difference and the unidimensional difference in the judgment-based choice task provide indirect evidence that participants tend to follow the certainty equivalent method when making a judgment-based choice, further research should continue to examine this possibility.

In summary, by examining brain activity, we supported our hypothesis that preferential choice cannot be characterized as a compensatory process with the aim of maximizing the overall value of a prospect in a conflict solution. Rather, it seems that preferential choice may be a non-compensatory process that is accompanied by strong conflicts. Together, these results tell a simple story: selection of the bet with the greatest overall value may appear to be an ideal way to solve conflict, but it does not capture the nature of risky decision making. Our study, from a perspective of decision conflict, attempted to address the issue of whether risky choices are based on a compensatory process. This perspective could potentially lead to a better understanding of the underlying mechanism of risky decision-making, which could present important directions for future research.
